# Skin marker combined with surface‐guided auto‐positioning for breast DIBH radiotherapy daily initial patient setup: An optimal schedule for both accuracy and efficiency

**DOI:** 10.1002/acm2.14319

**Published:** 2024-03-24

**Authors:** Jianjun Lai, Zhizeng Luo, Lu Jiang, Haili Hu, Chang Gao, Chuanfeng Zhang, Liting Chen, Jing Wu, Zhibing Wu

**Affiliations:** ^1^ Instiute of Intelligent Control and Robotics Hangzhou Dianzi University Hangzhou China; ^2^ Department of Radiation Oncology Zhejiang Hospital Hangzhou China

**Keywords:** breast radiotherapy, DIBH, setup efficiency, SGRT

## Abstract

**Background and purpose:**

By employing three surface‐guided radiotherapy (SGRT)‐assisted positioning methods, we conducted a prospective study of patients undergoing SGRT‐based deep inspiration breath‐hold (DIBH) radiotherapy using a Sentine/Catalys system. The aim of this study was to optimize the initial positioning workflow of SGRT‐DIBH radiotherapy for breast cancer.

**Materials and methods:**

A total of 124 patients were divided into three groups to conduct a prospective comparative study of the setup accuracy and efficiency for the daily initial setup of SGRT‐DIBH breast radiotherapy. Group A was subjected to skin marker plus SGRT verification, Group B underwent SGRT optical feedback plus auto‐positioning, and Group C was subjected to skin marker plus SGRT auto‐positioning. We evaluated setup accuracy and efficiency using cone‐beam computed tomography (CBCT) verification data and the total setup time.

**Results:**

In groups A, B, and C, the mean and standard deviation of the translational setup‐error vectors were small, with the highest values of the three directions observed in group A (2.4 ± 1.6, 2.9 ± 1.8, and 2.8 ± 2.1 mm). The rotational vectors in group B (1.8 ± 0.7°, 2.1 ± 0.8°, and 1.8 ± 0.7°) were significantly larger than those in groups A and C, and the Group C setup required the shortest amount of time, at 1.5 ± 0.3 min, while that of Group B took the longest time, at 2.6 ± 0.9 min.

**Conclusion:**

SGRT one‐key calibration was found to be more suitable when followed by skin marker/tattoo and in‐room laser positioning, establishing it as an optimal daily initial set‐up protocol for breast DIBH radiotherapy. This modality also proved to be suitable for free‐breathing breast cancer radiotherapy, and its widespread clinical use is recommended.

## 1 INTRODUCTION

Radiotherapy is an important component of the comprehensive treatment of breast cancer.^1^, ^2^, ^3^ Combining radiotherapy with surgery can reduce the rate of local and distant breast cancer recurrence, increase the rate of local control, and improve patient prognosis.^4^, ^5^ Radiation therapy for breast cancer inevitably exposes the organs and normal tissues surrounding the target area to some degree of radiation, and thus carries a risk of treatment toxicity for patients.^5^, ^6^ The deep inspiratory breath‐holding (DIBH) technique significantly reduces the mean dose of radiation to the heart and its substructures for left breast cancer radiotherapy, significantly reduces the maximal dose to the heart and the mean dose to the liver for right breast cancer radiotherapy, and improves the risk of organ toxicity to the patient's heart, lungs, and liver.^6^, ^7^, ^8^, ^9^, ^10^, ^11^

Breath‐controlled radiotherapy adopts optical surface imaging from the surface‐guided radiotherapy (SGRT) system, enables the dynamic monitoring of the patient's surface position and respiratory movement in real time before and during radiotherapy, offers non‐invasive and non‐irradiated treatment with high accuracy, and is widely used in DIBH radiotherapy for breast cancer.^12^, ^13^ Optical surface image‐guided initial patient positioning not only improves the accuracy of the positioning of radiotherapy for breast cancer, but also keeps the patient's skin free of markings and tattoos.^14^, ^15^, ^16^ Therefore, SGRT has been successfully used to deliver radiotherapy to various anatomical sites.

In May 2021, our department initiated a prospective study to enhance SGRT‐DIBH radiotherapy for breast cancer. Herein, by employing three SGRT‐assisted positioning methods, we aim to refine the daily initial positioning workflow and identify an optimal, efficient, and accurate protocol for the initial setup of SGRT‐assisted radiotherapy.

## 2 METHODS

We included patients who were subjected to breast radiotherapy (after breast‐conserving or radical surgery) between May 2021 and June 2023, showed good compliance, and completed the entire DIBH treatment process. A total of 124 patients with breast cancer received SGRT‐DIBH, 67 of whom exhibited left‐sided breast cancer and 57 of whom exhibited right‐sided breast cancer. The patients had an average age of 52.2 years (range: 22−72 years). All patients underwent consistent technology standards of immobilization, respiratory training, CT simulation, reference surface imaging, and treatment planning. The patients were randomized into three groups to assess the accuracy and efficiency of the initial daily setup in SGRT‐DIBH breast radiotherapy through a prospective comparative study. None of the patients were informed about the specific initial daily setup method assigned to their respective group. The enrolled patients were grouped as follows: Group A was subjected to the daily patient setup protocol of skin marker plus SGRT verification (39 patients), Group B underwent the daily patient setup protocol of SGRT optical feedback plus auto‐positioning (36 patients), and Group C was subjected to the initial patient setup protocol of skin marker plus SGRT auto‐positioning (49 patients).

### 2.1 Immobilization, CT simulation, reference surface, and planning

**FIGURE 1:**
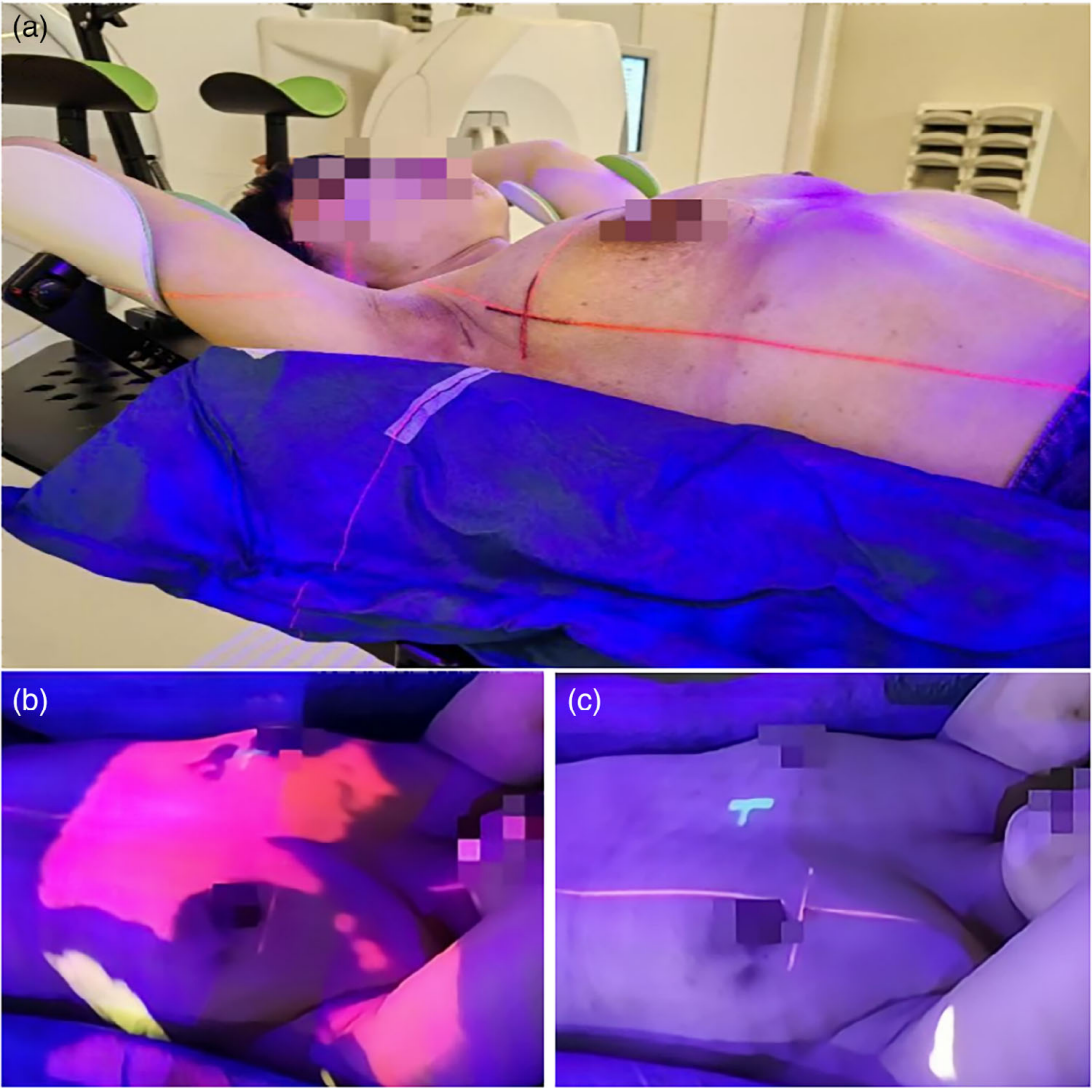
The figure (a) shows the patients undergoing skin marking and in‐room laser alignment setup in Group A and Group C, and also shows the trunk and arm immobilization methods for all patients in this study. The figure (b and c) shows the process of performing a coarse setup by means of SGRT optical projection feedback in Group B, a setup method without skin markers/tattoos. The figure (b) shows the patient before the setup is completed, and The figure (c) shows the patient with the setup completed. The red light projection on the skin is a 12 mm high position deviation, the yellow light projection on the skin is a 12 mm low position deviation, and no light projection is an acceptable position deviation (< 12 mm).

For patients with breast cancer undergoing SGRT‐DIBH radiotherapy, trunk immobilization was achieved through the creation of individualized negative pressure vacuum bag molds, while arm immobilization was facilitated using arm rests. Patients were trained to perform DIBH through thoracic breathing with a gating window width of 1.5−3 mm and a breath‐hold duration of ≥ 20 s; this was repeated more than three consecutive times using a laser‐based surface scanner (Sentinel, C‐RAD AB, Sweden) mounted at the CT end. After locating the isocenter of the plan target with the in‐room laser during free breathing, patients in groups A and C were marked with three crosshairs on the skin in accordance with the laser light (as shown in Figure 1a). Next, reference surface images for the SGRT were acquired under free breathing (FB) for the three groups of patients, and these were then sent to the optical‐based surface scanner (Catalyst, C‐Rad AB, Sweden) at the linear accelerator (LA) (Infinity, Elekta, Sweden) end.

The region of interest (ROI) for SGRT surface imaging at both the CT and LA ends included all anatomical structures from the patient's acanthiomeatal line to the lower edge of the costal arch, and the ROI was minimized as much as possible while still meeting the above requirements. Furthermore, CT images with a slice thickness of 3 mm were acquired using a CT simulator (Somatom, Siemens, Germany) for all patients under FB and DIBH; these images were then sent to the treatment planning system. Once received, the radiation oncologists performed clinical target volume (CTV) and OAR segmentation on the DIBH CT images, and the radiation physicists conducted DIBH treatment planning using 6 MV‐FFF of energy. The planning target volume (PTV) was defined as the expansion of CTV by 10 mm and its retraction to 5 mm subcutaneously. In this study, every patient's treatment plan was calculated using the Monte Carlo method on a Monaco 5.1 treatment planning system (Elekta, Sweden). These plans, after receiving approval from the radiation oncologists, were subsequently forwarded to the LA for plan validation before initiating treatment.

### 2.2 Initial setup, CBCT, and treatment implementation

#### 2.2.1 Initial daily setup protocol

**FIGURE 2:**
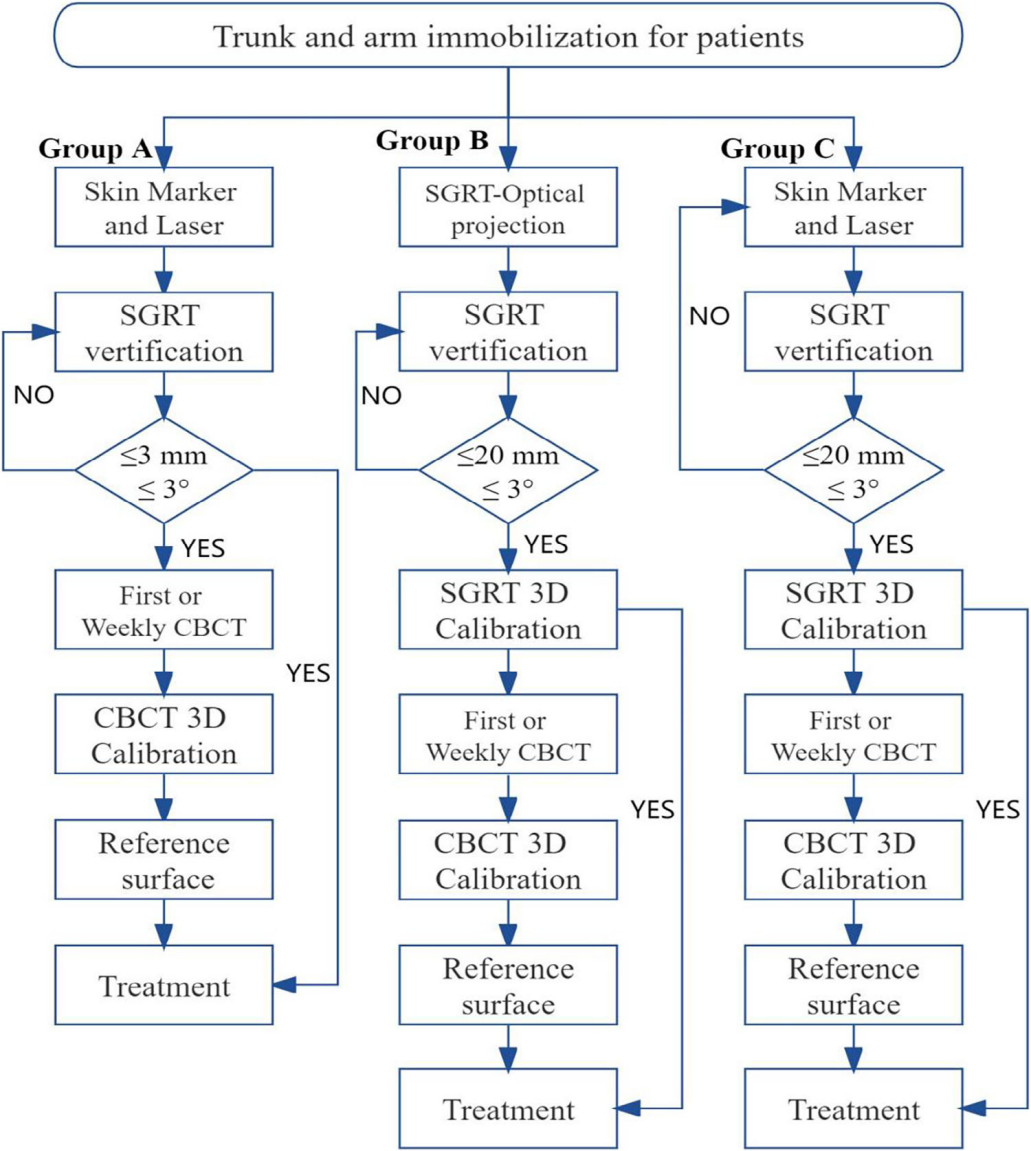
Schematic overview of the clinical workflow for the three groups in this study.

All patients were immobilized on the LA couch using individualized negative pressure vacuum‐bag molds for trunk immobilization and arm rests for arm immobilization (as shown in Figure 1a), followed by isocentric positioning using the three protocols in this study for the patients applying FB (as shown in Figure 2).

Group A: The patients in group A first underwent LA couch movement and body position adjustment to align the skin crosshair markers with the in‐room laser (as shown in Figure 1a). Subsequently, the position was verified using an optically based surface system with a standard translational deviation of ≤ 3 mm and rotational deviation of ≤ 3°. The patient's position was repeatedly adjusted as described above until the threshold was reached.

**FIGURE 3:**
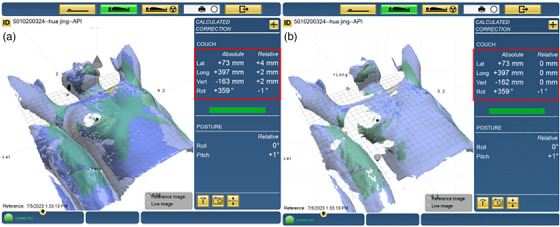
The figure shows the results of a patient in Group C before (a) and after (b) SGRT one‐key calibration of SGRT system.

Group B: The patients in group B initially used the feedback from the optical projection of the optically based surface scanner onto the patient's skin to guide their couch movement and positional adjustments (as shown in Figure 1b, c) until the translational and rotational deviations calculated by the optically based surface system were ≤ 20 mm and ≤ 3°, respectively. The translational setup error values were then sent to the LA couch for one‐key calibration (as shown in Figure 3).

Group C: The patients in group C first underwent LA couch movement and body positional adjustment to align the skin crosshairs markers with the in‐room laser (as shown in Figure 1a). Subsequently, the position was verified using an optically based surface system with a standard translational deviation of ≤ 20 mm and rotational deviation of ≤ 3°. The patient's position was repeatedly adjusted as described above until the threshold was reached, and the translational setup error values were then sent to the LA couch for one‐key calibration (as shown in Figure 3).

During the SGRT optical projection‐guided setup for patients in Group B, the therapists adjusted the LA couch and patient's body position under the guidance of three colored projections displayed by the optical system for positional calibration. Figures 1b and 1c illustrate the process of conducting a coarse setup using SGRT optical projection feedback in Group B, where red light projection indicates a high positional deviation of ≥ 12 mm, yellow light projection indicates a low positional deviation of ≥ 12 mm, and no light projection signifies an acceptable positional deviation (i.e., < 12 mm). In Groups B and C, while using optical projection or skin marker setup, the SGRT system calculated setup errors by aligning the patient's real‐time surface image with a reference surface image. After the completion of coarse setup, the SGRT system computed the final coarse setup error values (an example is shown in Figure 3a). Subsequently, the SGRT system transmitted the translational deviation (≤ 20 mm) to the LA couch for automatic calibration, and then displayed the post‐calibration setup error (an example is shown in Figure 3b).

#### 2.2.2 Cone‐beam computed tomography (CBCT) verification and reference surface acquisition

After completion of the initial setup protocol, CBCT under DIBH was first matched to the planning CT using automatic gray value matching with a clipbox including the whole length of the PTV, sternum, and vertebrae. The automatic result of the six degrees of freedom was visually evaluated and manually adjusted if the automatch was not considered acceptable. The three translational setup errors were calibrated using the LA couch, and the optical reference surface was acquired using the optical surface scanner under FB. All patients were required to perform the above procedure once per week. The patients in Groups A and C were required to re‐delineate the crosshair marker after completion of the first or weekly CBCT verification or if there was a setup error of > 3 mm or > 3°.

#### 2.2.3 Treatment implementation

All patients held their breath with a deep inspiration at the preset gating window and triggered the LA beam‐on using the Response gating interface. The patients were monitored in real time by the optical surface scanner during treatment for setup displacement.

### 2.3 Statistical analysis for setup error data and setup time duration

Kruskal–Wallis H tests were used to analyze the differences among the three groups of sample data, as our data were not normally distributed (as confirmed by the Shapiro–Wilk's test). SNK‐Q was used to check for two‐tailed differences between the three groups. The statistical tests and charts were generated using Origin 2021, and *p* < 0.05 was considered to indicate statistical significance.

## 3 RESULTS

### 3.1 Comparison of the accuracy of three initial setup protocols

Our study comprised setup errors in translation and rotation calculated from 496 fractions of CBCT registration (excluding the first CBCT) under DIBH in 124 patients, with 156 fractions in group A, 144 fractions in group B, and 196 fractions in group C. Setup errors in both translation and rotation were taken as displacement vector values for statistical analysis.

#### 3.1.1 Translational setup accuracy

**FIGURE 4:**
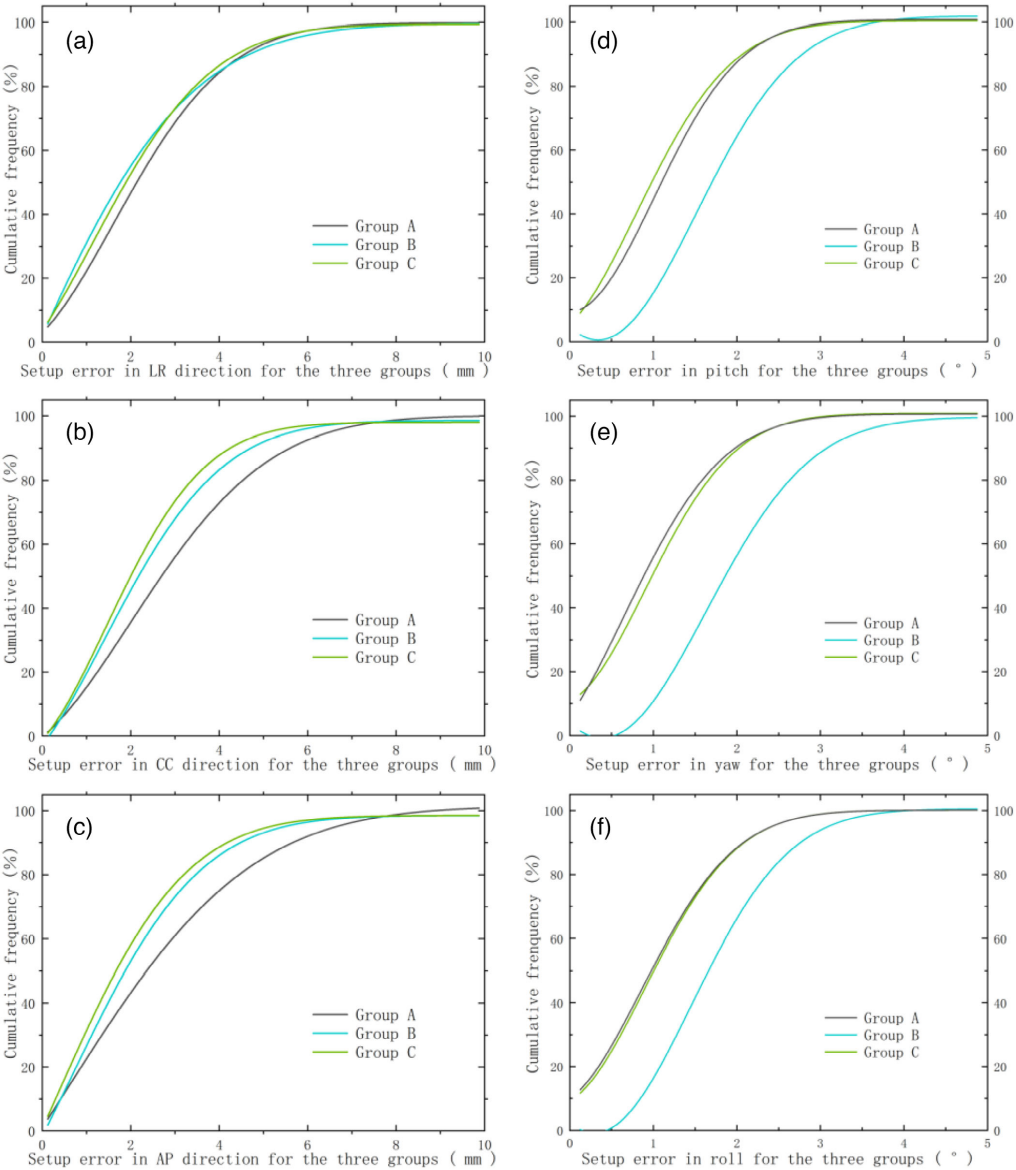
Cumulative frequency of the magnitude of the setup error vectors for the three groups of patients in the present study. (a‐c) Translational displacement vectors; (d‐f) Rotational displacement vectors. The cumulative frequency on the vertical coordinate represents the frequency of setup errors that are less than a certain value on the horizontal coordinate that occured in all CBCT verification fractions for each group.

As shown in Table 1, the mean and standard deviation of the translational setup error vectors in groups A, B, and C were small, with the highest values of the three directions observed in group A (2.4 ± 1.6 , 2.9 ± 1.8 , and 2.8 ± 2.1 mm); the percentage of abnormal setup error data ≥ 8 mm was < 1.5%. The three translation vectors in group C accounted for > 70% of the set‐up error within 3 mm (71.4%, 70.4%, 71.9%; *p* < 0.05). Figure 4a‐c shows the cumulative frequency distribution of the translational setup error, further demonstrating that the three initial patient setup protocols used in this study displayed good setup accuracy in translation, with patients in group A being slightly less accurate than those in groups B and C in the three translations—particularly in the craniocaudal (CC) and anterior‐posterior (AP) translations.

**TABLE 1:** Summary of the translation setup error data for the 124 patients with breast cancer included in this study, with three initial patient setup protocols.

	Directions	Group A (n = 39, f = 156)	Group B (n = 36, f = 144)	Group C (n = 49, f = 196)	*p*
Translation setup error (mm)	RL direction	2.4 ± 1.6	2.3 ± 1.8	2.3 ± 1.7	0.13
	CC direction	**2.9 ± 1.8**	2.6 ± 1.7	2.4 ± 1.7^*^, ^**^	< 0.05
	AP direction	**2.8 ± 2.1**	2.2 ± 1.7	2.3 ± 1.8^*^	< 0.05
Fractions with any excursion ∈[0, 3) mm, no. (%)	RL direction	102 (65.4)	102 (70.8)	**140 (71.4)** ^*^	< 0.05
	CC direction	83 (53.2)	98 (68.1)	**138 (70.4)** ^*^	< 0.05
	AP direction	99 (63.5)	**112 (77.8)**	141 (71.9)^*^	< 0.05
Fractions with any excursion ∈[3, 8) mm, no. (%)	RL direction	**52 (33.3)**	40 (27.8)	54 (27.6)	< 0.05
	CC direction	**71 (45.5)**	44 (30.5)	55 (28.1)^*^, ^**^	< 0.05
	AP direction	**56 (35.9)**	31 (21.5)	53 (27.1)^*^, ^**^	< 0.05
Fractions with any excursion ∈[8,∞) mm, no. (%)	RL direction	2 (1.3)	2 (1.4)	2 (1.0)	0.15
	CC direction	2 (1.3)	2 (1.4)	3 (1.5)	0.22
	AP direction	1 (0.6)	1 (0.7)	2 (1.0)	0.08

The most favorable values in setup error are marked in bold. AP, CC, and RL represent the anterior–posterior, cranio‐caudal, and right–left directions of the patients, respectively.

* Indicates Group C in comparison to Group A (*p* < 0.05).

** Indicates Group C in comparison to Group B (*p* < 0.05).

#### 3.1.2 Rotational setup accuracy

As shown in Table 2, the rotational errors in group B (1.8 ± 0.7°, 2.1 ± 0.8°, and 1.8 ± 0.7°) were significantly larger than those in groups A and C. With respect to the setup error vectors within 1°, the proportion of all three rotational vectors in groups A and C was > 37%, whereas the proportion of all three rotational vectors in group B was less than 13%. We observed that the percentage of abnormal setup data ≥ 3° in group B was the highest of all three groups, with the highest percentage of yaw rotation vectors (11.8%). Figure 4d‐f illustrates the cumulative frequency distribution of rotational setup vectors, with the curve in Group B exhibiting a downward deviation relative to Groups A and C that was in the low value range for the yaw vectors. Of the three setup methods adopted in the present study, groups A and C manifested better rotational setup accuracy, and the setup method implemented by patients in group B was significantly worse than those in the other two groups in terms of rotational setup accuracy.

**TABLE 2:** Summary of the rotational setup error data for the 124 patients with breast cancer included in this study, with three initial patient setup protocols.

	Directions	A (n = 39, f = 156)	B (n = 36, f = 144)	C (n = 49, f = 196)	*p*
Rotation setup error (mm)	Pitch	1.2 ± 0.7	**1.8 ± 0.7**	1.2 ± 0.7^**^	< 0.05
	Yaw	1.1 ± 0.8	**2.1 ± 0.8**	1.0 ± 0.6^**^	< 0.05
	Roll	1.2 ± 0.8	**1.8 ± 0.7**	1.3 ± 0.8^**^	< 0.05
Fractions with any excursion ∈ [0, 1)°, no. (%)	Pitch	59 (37.8)	18 (12.5)	**89 (45.4)** ^**^	< 0.05
	Yaw	**78 (50.0)**	12 (8.3)	92 (46.9)^**^	< 0.05
	Roll	**70 (44.8)**	17 (11.8)	86 (43.9)^**^	< 0.05
Fractions with any excursion ∈ [1, 3)°, no. (%)	Pitch	96 (61.5)	122 (84.7)	103 (53.1)^*^, ^**^	< 0.05
	Yaw	77 (49.3)	115 (79.9)	104 (53.1)^**^	< 0.05
	Roll	82 (52.6)	115 (79.9)	106 (54.1)^**^	< 0.05
Fractions with any excursion ∈ [3,∞)°, no. (%)	Pitch	**1 (0.7)**	4 (2.8)	3 (1.5)^*^	< 0.05
	Yaw	1 (0.7)	**17 (11.8)**	0 (0)^**^	< 0.05
	Roll	4 (2.6)	12 (8.3)	4 (2.0)^**^	< 0.05

The most favorable values in setup error are marked in bold. Roll, yaw, and pitch represent the rotation around the anterior–posterior, cranio‐caudal, and right‐left directions of the patients, respectively.

* Indicates Group C in comparison to Group A (*p* < 0.05).

** Indicates Group C in comparison to Group B (*p* < 0.05).

### 3.2 Comparison of the time consumed among the three initial setup protocols

**FIGURE 5:**
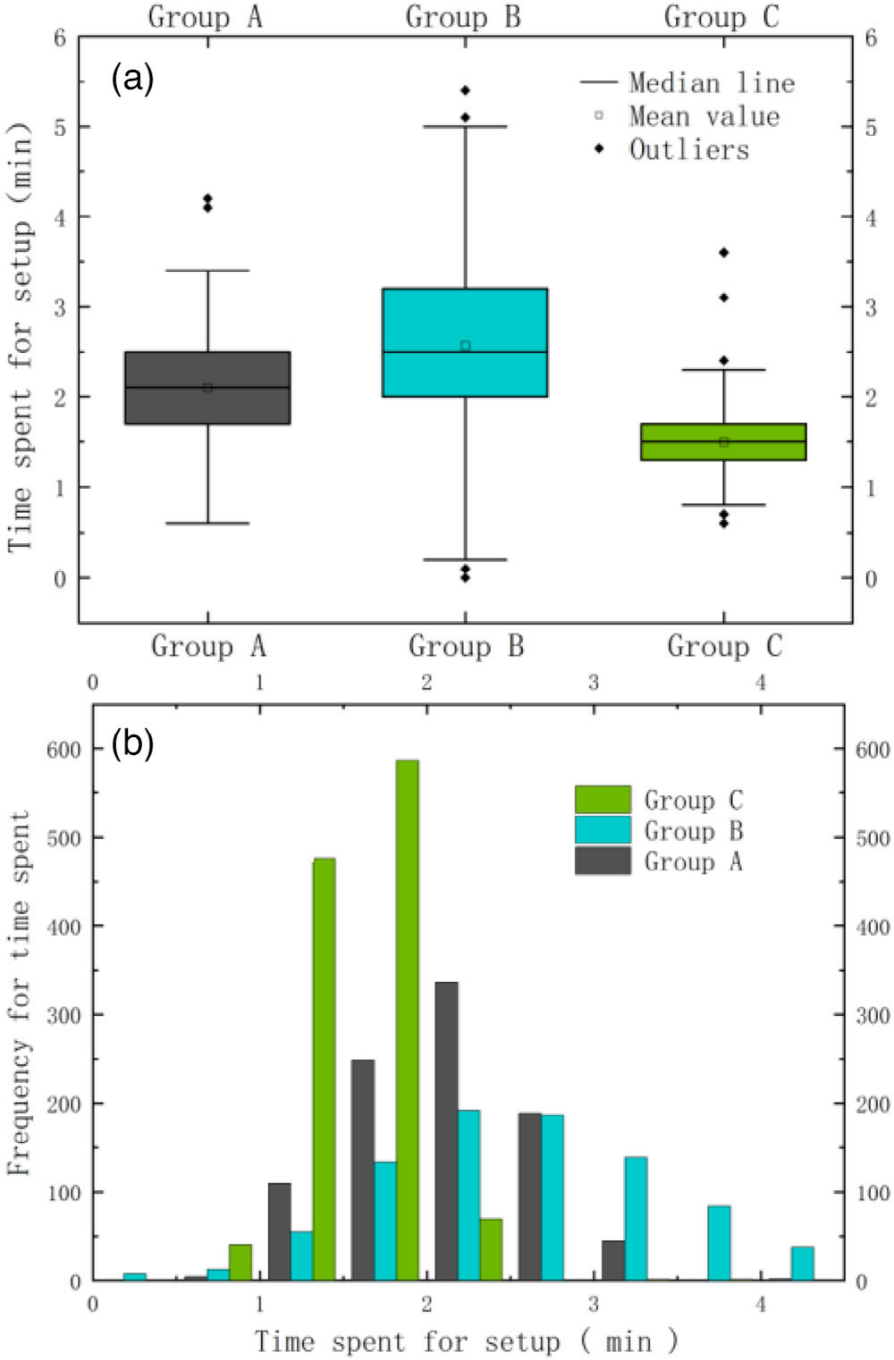
Box plots (a) and bar charts (b) of data comprising setup time consumed in this study.

The setup time was calculated as the time that the patient completes positional immobilization in the LA couch to the time the therapist leaves the LA room after completing all initial setup protocols, excluding the CBCT validation time and skin marker re‐delineation time. Table 3 shows the results of the time required for the three daily initial setup protocols, with Group C requiring the shortest time at 1.5 ± 0.3 min and Group B taking the longest time of 2.6 ± 0.9 min. The box plots (a) and bar graphs (b) in Figure 5 further illustrate the comparison of the three setup protocols used in this study. The optimal setup efficiency was achieved using the daily initial patient setup protocol with skin marker plus SGRT auto‐positioning.

**TABLE 3:** Summary of the setup time consumed for the 2976 fractions from 124 patients with breast cancer included in this study, with three initial patient setup protocols.

	Group A (n = 936)	Group B (n = 864)	Group C (n = 1176)	*p*
Time consumed (min)	2.1 ± 0.5	2.6 ± 0.9	**1.5 ± 0.3** ^*^, ^**^	< 0.05

The most favorable values in setup error are marked in bold.

* Indicates Group C in comparison to Group A (*p* < 0.05).

** Indicates Group C in comparison to Group B (*p* < 0.05).

## 4 DISCUSSION

Accurate positioning is a requirement for the delivery of precision radiotherapy.^17^ SGRT is a noninvasive, nonirradiated image‐guided radiotherapy technique that uses optical techniques to capture images of the patient's surface, and to detect and calibrate the patient's inter‐fractional setup errors to improve radiotherapeutic accuracy.^12^, ^13^, ^14^ In recent years, SGRT has become popular in breast cancer radiotherapy,^15^, ^16^ and several investigators have evaluated its use in the daily initial positioning of patients with breast cancer.^16^, ^18^, ^19^, ^20^, ^21^, ^22^, ^23^, ^24^, ^25^, ^26^, ^27^, ^28^, ^29^, ^30^, ^31^, ^32^ These studies allocated patients to two groups for comparative studies—skin markers/tattoos and non‐marking SGRT—and ascertained that the use of a non‐marking SGRT setup method improved the setup accuracy of the patient's radiotherapy. In the aforementioned studies, the authors only assessed the setup accuracy of the SGRT using the translational setup error, while few studies have focused on the rotational setup error.^16^, ^18^, ^19^, ^20^, ^21^, ^22^, ^23^, ^24^, ^25^, ^26^, ^27^, ^28^, ^29^, ^30^, ^31^, ^32^ Although the SGRT setup system can drive the LA couch to achieve automatic and rapid calibration for translational setup errors, the rotational setup error calibration requires repeated manual adjustments of the patient body position based on the optical projections and setup error data from the delayed feedback of the SGRT system. This has some potential drawbacks, such as its poor accuracy and time‐consuming nature. Kang et al.^33^ demonstrated the limitations of the SGRT system in correcting rotational offsets, but did not propose a solution. Therefore, in this prospective study, we attempted to combine SGRT with a skin marker/tattoo and laser setup method that focused on the setup errors of translation and rotation so as to generate a comparison with the non‐marking SGRT setup method. We also explored the optimal workflow of an SGRT‐assisted radiotherapy daily setup protocol that guarantees setup accuracy and work efficiency.

The results of the translational setup errors shown in Table 1 indicate that the SGRT system that calculates the setup error values and sends them to the LA couch for one‐key calibration achieves superior setup accuracy in the translational direction relative to the skin marker method. However, as shown in Table 2, Group B, with 33 abnormal data points from 20 CBCT fractions with setup error vectors > 3°, further demonstrated the limitations of the SGRT system in correcting for rotation shift. In our study, the setup method for Group B was a one‐key calibration in the direction of translation after a coarse setup by means of SGRT optical projection feedback; a setup method without skin markers/tattoos. When we employed this method to calibrate the rotational setup error, the calibration protocol was usually completed when the rotational error was manually calibrated to a value that was slightly below the threshold (3°) to ensure work efficiency. However, the slight involuntary movement of the patients after completion of the setup can cause abnormal data generation, allowing the rotational error to be > 3°. As shown in Tables 1 and 2, and Figure 4, a combination of skin markers and SGRT was used in this study to enhance both the calibration capability of skin markers for rotational setup errors and SGRT for translational setup errors, which resulted in the high accuracy of both the translational and rotational setup for group C patients. Kang et al.^33^ conducted a comparative study of the accuracy of breast radiotherapy using a skin marker setup and SGRT setup and found that in the translational direction, the mean setup errors for the skin marker method were 2.7 ± 1.6 , 2.0 ± 1.2 , and 2.1 ± 1.0 mm, while the mean setup errors for SGRT were 1.9 ± 1.2 , 2.9 ± 2.1 , and 1.9 ± 0.7 mm. The rotational setup errors for pitch, yaw, and roll without the SGRT approach were 0.32 ± 0.30°, 0.51 ± 0.24°, and 0.29 ± 0.22°, respectively, and with SGRT, the rotational errors were 0.30 ± 0.22°, 0.51 ± 0.26°, and 0.19 ± 0.13°, respectively. The translational errors for the SGRT group in the above study were similar to those of group C in this study, but the rotational errors were significantly lower than those of the three groups in this study. This difference may be because our study included DIBH patients, whereas the study of Kang et al. included FB patients. Another factor may be the differences between different positional immobilization techniques.

Comparisons of the time spent in non‐marker SGRT with the skin marker/tattoo set‐up have rarely been reported. Additionally, there have been significant inconsistencies in setup times,^19^, ^21^, ^32^, ^33^ which may be related to the fact that each radiotherapy unit entailed different positioning equipment, operating procedures, and technical standards. The results of this study with regard to the setup times showed that the shortest setup time (1.5 ± 0.3 min) was observed for the SGRT one‐key calibration after skin marker use and in‐room laser alignment in group C, which is significantly less than the non‐marker SGRT setup time reported by Kang et al.^33^ This variability can be explained by the difference in statistical criteria between our study and Kang et al.’s in terms of the time spent on setup. In this study, we did not consider the time spent acquiring CBCT images and setup verification. However, in their study, Kang et al. included the time required for setup verification and image acquisition. The longest setup time in the present analysis was for group B. This can be explained by the fact that the non‐marking SGRT optical projection and setup error feedback method required a longer amount of time to calibrate the rotational setup error. As shown in Table 3 and Figure 5, a combination of skin markers and SGRT was used in this study to achieve the highest daily initial patient setup efficiency.

## 5 CONCLUSION

Previous studies on daily initial setup methods for breast cancer DIBH radiotherapy did not focus on the effectiveness of the combination of skin markers and SGRT.^16^, ^18^, ^19^, ^20^, ^21^, ^22^, ^23^, ^24^, ^25^, ^26^, ^27^, ^28^, ^29^, ^30^, ^31^, ^32^, ^33^ In this prospective study, we demonstrated that SGRT one‐key calibration following skin marker/tattoo and in‐room laser positioning constituted an optimal daily initial setup protocol of breast DIBH radiotherapy that can account for translational and rotational setup errors and work efficiency. This modality is also suitable for use in FB breast cancer radiotherapy, and is thus recommended for widespread clinical use. In this study, we did not analyze the alignment results based on various SGRT ROIs, nor did we assess the baseline information on patient subgroups or differentiate between patients with right‐ or left‐sided breast cancer. These limitations will be addressed in future studies.

## AUTHOR CONTRIBUTIONS

The study was conceptualized and the methodology was set up by Jianjun Lai. Haili Hu, Chang Gao, Chuanfeng Zhang, Liting Chen, Jing Wu, and Lu Jiang analyzed the data; all authors were involved in the data interpretation. Jianjun Lai, Zhibing Wu, and Zhizeng Luo were the major contributors to the manuscript, which was thoroughly reviewed by Zhizeng Luo and Zhibing Wu. All authors read and approved the final manuscript.

## CONFLICT OF INTEREST STATEMENT

The authors declare that they have no conflicts of interest.

## ETHICS STATEMENT

The study was approved by the medical ethics committee of Zhejiang Hospital (No. 20210003J).
